# Awareness and its associated factors towards anesthesia and anesthetists’ among elective surgical patients in Debre Tabor Comprehensive Specialized Hospital, North Central Ethiopia 2021: Cross-sectional study

**DOI:** 10.1016/j.amsu.2021.102640

**Published:** 2021-07-29

**Authors:** Yewlsew Fentie, Tadelech Simegnew

**Affiliations:** Department of Anesthesia, College of Health Sciences, School of Medicine, Debre Tabor University, Debre Tabor, Ethiopia

**Keywords:** Patient, Awareness, Anesthesia, Anesthetist, Ethiopia

## Abstract

**Background:**

Patient awareness level of anesthesia and anesthetist is not well known in the study area which makes patients blind about the risk and benefits of anesthesia and the role of anesthetists'. This study aimed to assess elective surgical patients' awareness and its associated factors of anesthesia and anesthetists’ in Debre tabor comprehensive specialized Hospital, north-central Ethiopia, 2021.

**Method:**

Institutional based cross-sectional study was conducted on 367 patients who underwent for elective general surgery in Debre Tabor Comprehensive Specialized Hospital from December 15, 2020, up to May 15, 2021. Data were collected with a structured questionnaire of 13 items after translating the English version to the local language (Amharic). Descriptive statistics were expressed in percentage and presented with tables. Bivariable and multivariable logistic analysis were done to identify factors associated with the awareness level of patients on anesthesia and anesthetists. Statistical significance level was set at P < 0.05 with 95 % CI.

**Results:**

In this study, 25.1 % [95 % CI= (20.7–29.6)] of patients were adequately aware of anesthesia and anesthetist. Multivariable logistic analyses showed that male patients [AOR = 1.90; 95 % CI= (1.03–3.52)], level of education of secondary school [AOR = 3.20; 95 % CI= (1.07–9.61)] and collage and above [AOR = 4.75; 95 % CI= (1.73–13.06)], patients from Urban [AOR = 6.34; 95 % CI= (3.01–13.39)], and patients with previous anesthesia exposure [AOR = 3.43; 95 % CI= (1.76–6.69)] were more aware of anesthesia and anesthetist than their counterparts.

**Conclusion:**

The awareness level of patients about anesthesia and anesthetists in this study was poor. Sex, residency, educational level, and previous anesthesia exposure were factors associated with patients’ awareness level of anesthesia and anesthetists.

## Highlights

• Awareness of patients about anesthesia improves clinical service.
• Awareness of patients about anesthesia was poor in DCSH.
• Sex, residency, and previous anesthesia exposure are associated with awareness.
• Awareness creation strategies are required to improve awareness levels.

## Acronyms and Abbreviations

AOR: Adjusted Odds Ratio
CI: Confidence Interval
COR: Crude Odds Ratio
DTCSH: Debre Tabor Comprehensive Specialized Hospital.

## 1 Introduction

Patient awareness is an important component of anesthesia care service that helps patients to know the risk and benefits of anesthesia and the scope of practice of anesthetists. It also increases patient preference-based anesthesia services [[1], [2], [3]]. The awareness level of patients in developed countries ranges from 18% to -89 % [4].

Currently, Anesthesia is advancing in the knowledge of medical professionals, pharmaceutical drugs, and technological equipment to deliver the safest anesthesia care service for patients [5,6]. But, awareness and image of the community and patient about anesthesia as well as the role of anesthetists is always a problem especially in developing countries [[7], [8], [9]].

Patient awareness about anesthesia and the role of anesthetists can be influenced by patient-related barriers such as poor educational level and anesthesia exposure, as well as health professionals’ related problems such as deficit in community awareness creation and patient education [3,10,11].

Developing counties such as Ethiopia, have a shortage of anesthesia providers with limited service providers and limited resources to deliver anesthesia [[12], [13], [14]]. Also, a low level of patient education strategies and reduced governmental focuses on awareness creation with media hinders the awareness level of the patients about anesthesia and the role of anesthetists [12,15,16].

Even though few studies in anesthesia have assessed patient awareness about anesthesia and anesthetists, those are restricted to specific areas of surgical patients (1). Patient awareness about anesthesia and anesthetists and associated factors remains largely undiscovered (1).

It is very important to identify areas in which patients lack awareness about anesthesia that can help to improve the knowledge of patients. Therefore, the purpose of this study was to assessed awareness level and its associated factors of anesthesia and anesthetists among elective surgical patients.

## 2 Methods and materials

### 2.1 Study design, area, population, and period

This cross-sectional was conducted in DTCSH, which is a public hospital established in 1934 and located in the south Gondar zone of Amara regional state at 667 km Northwest of Addis Ababa, the capital city of Ethiopia. It is 97 km to the southwest of Bahir Dar, the capital city of Amara regional state. According to the 2007 census, the total population of this town was 155,596. It has a latitude and longitude of 11051N3801′E11.8500 N 38.0170E with an elevation of 2706 m (8878 ft) above sea level [17]. The hospital provides surgical and anesthesia services with six operation theatres. The study was conducted among patients who met inclusion criteria and underwent elective general surgery from December 15, 2020, up to May 15, 2021.

The study is registered at https://www.researchregistry with researchregistry6930. Also, it is reported according to STROCSS criteria [18].

### 2.2 Inclusion and exclusion criteria

#### 2.2.1 Inclusion criteria

Elective Surgical patients with the age of 18 and above were included in the study.

#### 2.2.2 Exclusion criteria

Patients with Cognitive dysfunction or other inabilities to finish the interview (communication or hearing impairment), Very seriously ill patients who cannot communicate, and Patients who were operated in the minor operation room were excluded from the study.

#### 2.2.3 Dependent variable

Surgical Patients’ Awareness towards Anesthesia and Anesthetist.

#### 2.2.4 Independent variables

Age, sex, marital status, Residency, Job, Educational status, and previous anesthesia exposure were the independent variables.

### 2.3 Sample size and sampling technique

#### 2.3.1 Sample size

The sample size was determined by using single proportion population formula taking the proportion from a study done at Black Lion Specialized hospital, Ethiopia with an overall knowledge level of 31.8 % [1], and the sample size was calculated by using a 95 % confidence interval and 5 % margin of error. The sample size was determined using the following formula.

$$n={\left(Z_{a/2}\right)}^{2}P(1-P)/d^{2}$$

whereas; n = sample size Z = confidence interval (1.96) P = estimated prevalence (0.647).

d = margin of sampling error to be tolerated (0.05)

$$n=\;\frac{{\left(1.96\right)}^{2}0.318\left(1-0.318\right)}{{\left(0.05\right)}^{2}}=333.26\sim 333$$

Constituting, the values into a formula, gives n = 333.

By considering a 10 % non-respondent rate the final sample size is 367.

#### 2.3.2 Sampling technique

All consecutive surgical patients who met the inclusion criteria were sampled till the intended sample size was achieved.

### 2.4 Data collection instrument and procedures

Patients’ awareness level was assessed with a structured questionnaire taken from previous studies in Ethiopia and Pakistan after the necessary correction was done to the clinical setting (1, 2).

The questioner has a total of 13 questions in which six questions assess the awareness level of patients towards anesthesia and seven questions measure awareness of patients about anesthetists. The English version of the tool was found with the internal consistency of Cronbach's alpha 0.79. Also, it was translated to the Amharic version of the local language with language experts and back again to English to ensure the proper translation with the other two language experts. Finally, the language expert committee argued with minor modifications. The internal consistency of the Amharic version of the tool was measured and was found (Cronbach's alpha = 0.82) which is higher than the English version. The data was collected by three BSc anesthetists when patients came to the pre-anesthesia clinic before anesthetists perform a pre-operative assessment.

#### 2.4.1 Data quality assurance

After training was given to data collectors, data were collected and properly filled in the prepared format. The supervision was made throughout the data collection period to make sure the accuracy, clarity, and consistency of the collected data.

#### 2.4.2 Ethical consideration

The ethical clearance to conduct the study was obtained from Debre Tabor University and additionally, the written informed consent was taken from every patient who was participated in the study before the start of the interview after telling them about the objective of the study. Confidentiality and secrecy were ensured.

#### 2.4.3 Data entry, analysis, and interpretation

The data were entered into Epidata version 4.2 and exported to SPSS version 20 for analysis. Descriptive statistics were carried out, and both bi-variable and multivariable logistic regression analyses were used to identify factors associated with the awareness level of patients about anesthesia and anesthetists. Hosmer-Lemeshow test of goodness of fit was performed to check the appropriateness of the analysis model. Variables with a p-value of less than <0.2 in the bivariable logistic analysis were fitted into a multivariable logistic regression analysis and a multicollinearity test was done. Both crude odds ratio (COR) in bivariable logistic regression and adjusted odds ratio (AOR) in multivariable logistic regression with the corresponding 95 % confidence interval were calculated to show the strength of association. In multivariable logistic regression analysis, variables with a p-value <0.05 were considered statistically significant.

### 2.5 Operational definition

**Aware of anesthesia and anesthetists:** When 50 % or more awareness questions about anesthesia and anesthetists are answered correctly by patients (1).

**Not aware of anesthesia and anesthetists:** When less than 50 % of awareness questions about anesthesia and anesthetists are answered by patients (1).

## 3 Result

### 3.1 Socio-demographic characteristics of patients

A total of 367 elective surgical patients were involved in the study, but 362 patients have completed the survey with a response rate of 98.6 %. Five incomplete questionnaires were excluded from the data. The majority of patients were with the age of (25–39, 32.1 %) and male gender (217, 59.9 %). A large number of patients were from rural areas (210, 58.0 %) and illiterate (124, 34.3 %) (Table 1).

**Table 1:** Socio-demographic characteristics of patients (n = 362).

Variables	Frequency (n)	Percentage (%)
**Age(years)**	75	20.7
18–24	99	27.3
25–39	116	32.1
50 and above	72	19.9
**Sex**		
Male	217	59.9
Female	145	40.1
**Marital status**		
Single	109	30.1
Married	211	58.3
Widowed	20	5.5
Divorced	22	6.1
**Residency**		
Urban	152	42.0
Rural	210	58.0
**Educational Level**		
Illiterate	124	34.3
read and write	103	28.5
primary school	34	9.4
secondary school	31	8.6
college and above	70	19.3
**Job**		
No occupation	13	3.6
House wife	68	18.8
Farmer	139	38.4
Student	52	14.3
Government employ	43	11.9
Private employ	47	13.0
**Previous anesthesia exposure**		
Yes	78	21.5
No	284	78.5

### 3.2 Awareness level of patients about anesthesia and anesthetist

The mean patients' awareness level about anesthesia and anesthetist was 4.7 with a standard deviation of (SD = 3.5). The majority of patients; 75 (20.7 %) scored a mean of 3 while about 5 (1.4 %) of patients were aware of all awareness questions. Only a few numbers 91 (25.1 %) [95 % CI= (20.7–29.6)] of patients were adequately aware of anesthesia and anesthetist. Anesthesia is necessary for surgery 362 (100.0 %) and anesthetists are responsible to administer anesthesia259 (71.5 %) were the most correctly responded awareness questions. While the type of anesthetic drugs used to deliver anesthesia and roles of anesthetists were aware of few respondents (Table 2).

**Table 2:** Awareness level of patients about anesthesia and anesthetist.

Variables	Correctly answered No (%)	Wrongly answered No (%)
Awareness about anesthesia		
Do you think anesthesia is necessary for surgery?	362 (100.0 %)	0 (0.0 %)
What are the different types of anesthesia?	94 (26.0 %)	268 (74.0 %)
Which anesthetic drugs are used during surgery?	72 (19.9 %)	290 (80.1 %)
Two hours before surgery what foods are allowed?	92 (25.4 %)	270 (74.6 %)
What are the complications of anesthesia?	77 (21.3 %)	285 (78.7 %)
If patients have coexisting disease/smoker or alcoholic risk of complication increases?	211 (58.3 %)	151 (41.7 %)
**Awareness about anesthetists**		
Who is responsible for administering anesthesia?	259 (71.5 %)	103 (28.5 %)
Who determines whether the patient is fit for anesthesia or surgery?	99 (27.3 %)	263 (72.7 %)
Who decides if the patient can eat before surgery?	95 (26.2 %)	267 (73.8 %)
What do you think about the role of the anesthetist in the operation room?	108 (29.8 %)	254 (70.2 %)
Who estimates and transfuses blood when needed during operation?	88 (24.3 %)	274 (75.7 %)
Who makes sure the patient recovers smoothly after surgery?	93 (25.7 %)	269 (74.3 %)
In which of the following roles are anesthetists involved?	48 (13.3 %)	314 (86.7 %)

### 3.3 Factors associated with an awareness level of patients to anesthesia and anesthetist

The bivariable logistic analyses showed that, sex, educational level, residency and previous anesthesia exposure were significantly associated with awareness level of patients about anesthesia and anesthetist. Also, in multivariable logistic recreation, sex [AOR = 1.90; 95 % CI= (1.03–3.52)], level of education [AOR = 3.20; 95 % CI= (1.07–9.61)] and [AOR = 4.75; 95 % CI= (1.73–13.06)], residency [AOR = 6.34; 95 % CI= (3.01–13.39)], and previous anesthesia exposure [AOR = 3.43; 95 % CI= (1.76–6.69)] were significantly associated with awareness level.

According to the result, male patients were almost 1.9 times more likely to be aware of anesthesia and anesthetists than females. Likewise, patients with the educational level of college and above as well secondary school were 4.75 vs. 3.20 times more likely to be aware than illiterates respectively.

Also, the likelihood of being from urban was 6.34 times more aware than being from a rural while; those patients who were exposed to anesthesia previously were 3.43 times more to be aware of their counterparts (Table 3).

**Table 3:** Factors associated with an awareness level of patients to anesthesia and anesthetist (n = 362).

Variables	Awareness level		Crude odds ratio	Adjusted odds ratio	p-value
	Aware	Not aware	(95 % CI)	(95 % CI)	
**Sex**					
Male	62 (28.6 %)	155 (71.4 %)	1.89 (1.02,3.52)	1.90 (1.03,3.52)	0.041*
Female	29 (20 %)	116 (80.0 %)	1	1	
**Level of education**					
Illiterate	12 (9.2 %)	119 (90.8 %)	1	1	
read and write	15 (14.6 %)	88 (85.4 %)	1.05 (0.41,2.71)	0.91 (0.36,2.29)	0.83
primary school	10 (29.4 %)	24 (70.6 %)	2.13 (0.68,6.65)	1.77 (0.59,5.31)	0.310
secondary school	12 (38.7 %)	19 (61.3 %)	4.05 (1.26,13.04)	3.20 (1.07,9.61)	0.038*
college and above	42 (66.7 %)	21 (33.3 %)	5.90 (2.07,16.85)	4.75 (1.73,13.06)	0.003*
**Residency**					
Urban	74 (50.3 %)	73 (49.7 %)	6.14 (2.89,13.05)	6.34 (3.01,13.39)	0.00*
Rural	17 (7.9 %)	198 (92.1 %)	1		
**Previous anesthesia exposure**					
Yes	33 (41.8 %)	46 (58.2 %)	3.08 (1.56,6.06)	3.43 (1.76,6.69)	0.00*
No	58 (20.5 %)	225 (79.5 %)	1		

* = p-value<0.05, 1 = reference.

## 4 Discussion

Lack of awareness about anesthesia and anesthetist is a great problem in anesthesia practice (19, 20). However, it can be improved by delivering health information regarding anesthesia and anesthetist with verities of health information delivering systems of the ministry of health or patient education by health professionals [20].

This study revealed that only 25.1 % of patients were adequately aware of anesthesia and anesthetist. This finding is lower than a study done in Britain, Hong Kong, Korea, and Ethiopia at Black lion specialized hospital anesthetist [1,19,21,22]. The possible explanation for variation might be due to study participants' differences in information delivery about anesthesia and the accessibility of anesthesia professionals.

The highest level of awareness was seen on “anesthesia is necessary for surgery and anesthetists are responsible to administer anesthetic drugs”. This result is nearly similar to a study done in Ethiopia and India [1,23]. Also, the lowest level of awareness was seen on “the type of anesthetic drugs used to deliver anesthesia and the roles of anesthetists”. This is similar to a study done in Pakistan which showed patients were less aware of the roles of anesthetists [2].

In this study male patients were more associated with awareness anesthesia and anesthetists than females. This finding was also similar to studies done in India and Ethiopia [1,24]. Also, regarding the residency of the patients, this study depicted that living in Urban was more associated with a high awareness level than rural. This result was in-lined with studies done in Ethiopia (1). The possible explanation for this might be, coming from urban areas are expected to be more informed about medical issues and get access to health information than murals.

This study showed that there is a positive association between level of education and awareness level about anesthesia and anesthetists in which patients with higher educational levels were more aware of it. This result is repeated in a study done in Ethiopia, Pakistan, India, Nigeria, and Saudi Arabia [1,2,[24], [25], [26]]. The possible explanation for this might be education might improve the awareness level of medical knowledge.

Regarding previous anesthesia exposure of the patients, this study depicted that patients who were previously exposed to anesthesia were more associated with the awareness level of anesthesia and anesthetists than none exposed to anesthesia. This was similar to studies done in Pakistan, India, and Australia [2,24,27]. But this result is a reverse in a study done in Ethiopian at Tikur Anbessa Specialized Hospital (1). The possible explanation for this variation might be differences in study populations' location, literacy level, and access to medical services or information.

## 5 Conclusion

The awareness level of patients about anesthesia and anesthetists in this study was poor. Sex, residency, educational level, and previous anesthesia exposure were factors associated with patients’ awareness level of anesthesia and anesthetists. So, it is suggested that the ministry of health and health professionals must plan strategies of awareness creation of patients about anesthesia and anesthetists.

### 5.1 Strength of the study

Study participants were homogeneous and data was collected with few none response rates.

### 5.2 Limitations of the study

The limitation of this study was first, it was conducted at a single health organization which might not generalize other organizations' patient awareness levels. Second, a high percentage of illiterate patients might affect the strength of the study. Additionally, randomization and blinding were not applied.

## Availability of data and material

The data of this study will be available from the corresponding author on reasonable request.

## Declaration of competing interest

The authors declare there is no competing interest in this work.

## Provenance and peer review

Not commissioned, externally peer-reviewed.

## Declaration of competing interest

Nothing to declare.

## Registration of research studies

Name of the registry: http://www.researchregistry.com.

Unique Identifying number or registration ID: researchregistry6930.

Hyperlink to your specific registration (must be publicly accessible and will be checked): https://www.researchregistry.com/browse-the-registry#home/?view_2_sort=field_21|asc.
